# Surgical Site Infection Post-appendectomy in a Tertiary Hospital, Jeddah, Saudi Arabia

**DOI:** 10.7759/cureus.16187

**Published:** 2021-07-05

**Authors:** Mohammed I Koumu, Abdulkarim Jawhari, Saleh A Alghamdi, Mutasem S Hejazi, Ali H Alturaif, Saleh M Aldaqal

**Affiliations:** 1 Surgery, Trauma Center, King Saud Medical City, Riyadh, SAU; 2 College of Medicine, King Abdulaziz University, Jeddah, SAU; 3 General Surgery, King Abdulaziz University Hospital, Jeddah, SAU

**Keywords:** surgical site infections, laparoscopic appendectomy, ssi, appendectomy, appendectomy variants

## Abstract

Background and purpose: Appendectomy considered at the top of emergency surgical procedures worldwide, and surgical site infection (SSI) is not an uncommon complication postoperatively. Many factors may be contributed to SSI occurrence; either during preoperative, intraoperative, or postoperative periods. No recent studies focusing on SSI post-appendectomy and the related factors in our region. So, we aim to find the prevalence and detect the factors that may lead to SSI in post-appendectomy patients at King Abdulaziz University Hospital (KAUH) between 2013 and 2017.

Methods: This is a retrospective chart review study. Data were collected by data collection sheet from (KAUH) patient’s database, as we include: patients' demographics, blood investigations, operation details, co-morbidities, and hospitalization time. All patients who underwent appendectomy between 2013 and 2017 were included. We used frequencies, Mann-Whitney U test, and binary logistic regression tests for data analysis.

Result: SSI post-appendectomy was found in 31 patients out of 433. SSI was statistically significant related more with an open technique of appendectomy (p=0.0001), longer duration of the surgery (p=0.0001), perforated type of appendicitis (p=0.002), more hospitalization time (p=0.0004), postoperative lab results of high WBC count (p=0.004), and low albumin (p=0.011). Other factors including demographics and clinical characteristics, intraoperative, perioperative, and hemoglobin level showed no significant relations.

Conclusion: Controlling the high rate of SSI by using the optimal technique of approach, decreasing the duration of the surgery, and early intervention may help more in reducing SSI post-appendectomy. Taking into consideration the other perioperative factors will lead to better outcomes for the patients.

## Introduction

Appendectomy is considered very popular and standard management in many cases of acute appendicitis. At the same time, it is one of the top emergency surgeries in the world these days [1]. There are mainly two surgical approaches regarding appendectomy, laparoscopic and open. The laparoscopic approach becomes more preferable in the practice rather than the classic open approach [2] because of its better outcomes in decreasing length of stay, postoperative pain, the possibility of getting wound infection and recovery time [3]. Perforated and gangrenous appendicitis are possible complications that may happen, which are even harder to treat and have a worse prognosis than simple uncomplicated appendicitis [4].

Different studies presented how common is appendectomy. The incidence of appendicitis or appendectomy according to Ferris et al., in their review of the population-based study was more stable in western countries than in newly industrialized countries where an increasing pattern of incidence was observed [1]. Mean pooled annual incidence per 100,000 people since 2000 was found to be 100 in Northern America, 113 in Northern Europe, 112 in Southern Europe, 105 in Eastern Europe, 151 in Western Europe, 140 in Oceania, 160 in Turkey, 97 in Taiwan, 28 in Nigeria and 15 in South Africa. A large population sample study in Bergamo, Italy, showed that 16,544 patients were presented with acute appendicitis from 1997 to 2013, and 94.7% of them underwent appendectomy procedures [5]. At the local level, in Riyadh, 852 patients presented to King Khalid University Hospital between 1999 and 2003 underwent appendectomy [6]. While in the same hospital, laparoscopic appendectomy was done among 100 children between 1999 and 2003 [7]. Another previous study at King Abdulaziz University Hospital (KAUH) in 2003 showed 124 females patients presented in the emergency department, where 103 (83.1%) of them underwent appendectomy [8].

Surgical site infection (SSI) counts as an undesirable and critical issue that leads to a high rate of morbidities and mortalities postoperatively [9]. According to the Centers for Disease Control and Prevention (CDC), it is defined as infections that affect the surgical wounds (skin), underling fascia, spaces, or organs postoperatively. However, the beginning of manifestations can happen within 30 to 90 days - according to the type of surgery. As we are going through the exact clinical picture and the causative organisms for SSI, patients’ normal flora is believed to have the upper hand in SSI etiologies. While multidrug-resistant micro-organisms were found in the majority of the cases of SSI after an appendectomy, Escherichia coli, Staphylococcus aureus, and Pseudomonas aeruginosa are the most common ones [10,11].

A combination of sterile techniques includes prophylactic antibiotics, antiseptic usage, taking care of patients at risk, and postoperative care will assist in SSI prevention. Antiseptics are highly effective in SSI prevention preoperatively, as it removes the skin’s normal flora easily, subsequently preventing their arrival to the deeper tissues after incision [12].

The mean point, appendectomy is considered a contaminated procedure that has a significantly high percentage of SSI rate that can reach up to 40% after a surgical procedure [13]. A systemic review done on 35 studies found that the overall rate of infection for open appendectomies reached 17.9% (CI: 95% with 10.4-25.3 infections/100 procedures) and a percentage of 8.8% (CI: 95% with 4.5-13.2 infections/100 procedures) of laparoscopic appendectomy [14]. Danwang et al., in their systematic review with meta-analysis including more data from 49 different countries, showed the overall incidence of 7% (CI: 95% with 1-17.6 infections/100 procedures) [11].

In Saudi Arabia, an old project with a period of 12 months was done on 1,770 wounds and has shown a rate of infection of 9.4% postoperatively with a percentage of distribution of 55.6 for general surgery. A female to male ratio of 4:1, and age ranging from four days to 90 years old, with prophylaxis of antibiotics given to all patients who underwent an appendectomy [15]. Results of another study on 227 patients who underwent an emergent appendectomy showed that the laparoscopic method was done on 108 of the patients and the open method on 119. The laparoscopic procedure group showed no infection. While the open group showed nine who had wound infection with a 7.6% rate of infection [16].

However, no recent studies focusing on the risk factors of getting SSI with the appendectomy procedures are available in our region. So, we aim to find the prevalence and detect the potential risk factors that may lead to SSI in post-appendectomy patients at KAUH between 2013 and 2017.

## Materials and methods

This study was approved by the Unit of Biomedical Ethics Research Committee of King Abdul-Aziz University, faculty of medicine (Reference No. 589-18).

This is an observational retrospective chart reviewing the medical record where we aim to find the prevalence and detect the risk factors that may lead to SSI in post-appendectomy patients at KAUH, Jeddah from 2013 to 2017. KAUH is considered one of the biggest educational and referral hospitals in the western region of Saudi Arabia. It includes more than 1,000 beds and has more than 2,800 admissions per year to the surgical department.

We include in the study all patients who underwent appendectomy within the five years from 2013 to 2017 without any age limits. Except for those patients who had colon procedures that include the removal of the appendix, or had a major procedure that is not related to the appendix but appendectomy was done incidentally, they have been excluded from the study because of the higher risk for SSI. CDC criteria of SSI were used to define patients with the diagnosis.

We collect the data from the patients' electronic medical files at the hospital by using a data collection sheet. We include any available: patients' demographics (gender and age), operation details (using prophylactic antibiotics, the urgency of the procedure, American Society of Anesthesiologists [ASA] classification of preoperative risk assessment, type of skin preparation, preoperative shaving, approach type [either open or laparoscopic], duration of the procedure, and wound infection category), the mean value of three days postoperative patients' readings of (white blood cells count [WBC], hemoglobin [Hb] and albumin level), patients' status for hypertension (HTN), diabetes mellitus (DM), having SSI within 30 days post-appendectomy, histopathological type of the appendix specimen (simple acute appendicitis, gangrenous appendicitis, perforated appendicitis, suppurative appendicitis, no pathology, or other findings) and hospitalization time (HT).

The collection of the patients' data was kept confidential, saved in a secured computer, and then all of the data will be deleted after finishing the study.

Data were analyzed using IBM SPSS statistics version 20 (SPSS Inc., Chicago, IL, USA). Chi-square test or Fisher's exact test were used to examine the relation between qualitative variables as appropriate. For quantitative data, the comparison between two groups was done using the Mann-Whitney U test and the independent t-test for the relation between qualitative and quantitative factors. Binary logistic regression was applied to determine risk factors associated with SSI (dependent variable). A p-value < 0.05 was considered significant.

## Results

In the five years from 2013 to the end of 2017, 433 of the patients matched our selection criteria and underwent an appendectomy. Their age ranged from eight days to 85 years old, with a mean [SD] age of 25 [14.54] years. The majority of them were male patients n(%) 253(58.4) and 180(41.6) were female patients. They had 26 different nationalities, where Saudis had the biggest proportion of them by 62.4%, while non-Saudis represent 37.6% of the total. Table 1 summarizes patients' demographics and clinical characteristics.

**Table 1:** Demographic and clinical characteristics among studied patients DM - diabetes mellitus

Demographic and clinical characteristics	The studied patients (No=433)
Age (year)	
Mean±SD	25.05±14.54
Median (range)	22 years (8 days–85 years)
Gender	
Male	253 (58.4%)
Female	180 (41.6%)
Hypertension	
No	408 (94.2%)
Yes	25 (5.8%)
DM	
No	409 (94.5%)
Yes	24 (5.5%)
Both hypertension and DM	
No	419 (96.8%)
Yes	14 (3.2%)
Blood types	
A+	150 (34.6%)
AB	23 (5.3%)
B+	61 (14.1%)
B-	3 (0.7%)
A-	9 (2.1%)
O+	165 (38.1%)
O-	22 (5.1%)

Among the 433 patients who underwent appendectomy, SSI was detected in 31 patients, the association between the different factors and the SSI, statistical analysis showed no significant relationship with the demographic data of the patients and their clinical characteristics; mean age (p=0.23), gender of the patient (p=0.67), having hypertension (p=0.87), DM (p=0.82) or both (p=0.29), and blood grouping (p=0.06).

Giving prophylactic antibiotics also was not significantly associated with SSIs (p=0.19) except for Cefuroxime specifically given preoperatively had the benefit of decreasing the risk of infection in these patients (p=0.041). All other used antibiotics were not associated with the incidence of SSIs in a significant way (p≥0.05). The number also, whether it is only a single agent or multiple antibiotics that were used, was not a significant factor (p=0.403).

However, regarding factors that are related to the operation, a significant relation was found with the type (p=0.0001) and duration (min.) (p=0.0001) of appendectomy where the laparoscopic approach was less associated with the SSIs than the open appendectomies, 2.9% and 12.6% out of all patients respectively. Also, as the procedure takes a longer time, the risk of SSI will be increased. But on the opposite side, a non-significant relationship was found between the SSI and ASA classification, preoperative shaving, type of antiseptic used for preparation, the urgency of the operation, converting to open appendectomy, and wound infection category (p≥0.05). Histopathological type of appendicitis is another important factor, where SSI was found to be associated in particular with 45 (10.4%) cases of perforated appendicitis (p=0.002). However, other complicated types of appendicitis, simple appendicitis, or even normal appendix were not significantly associated with SSI. Table 2 summarizes the association and distribution of all factors among the patients with and without SSI.

**Table 2:** Univariate analysis for risk factors associated with SSI and binary logistic regression for the significant factors *significant difference; OR: Odds Ratio (CI: Confidence Interval); DM - diabetes mellitus; ASA - American Society of Anesthesiologists; SSI - surgical site infection

Risk factors	Studied patients (No=433)		Test of significance	P-value
				With SSI	Without SSI	OR (95% CI)
	(No=31)	(No=402)		
	Age (year)	24.42±18.14		25.10±14.26	Mann Whitney U test=1.20	0.23
Gender				
Male	17 (54.8%)	236 (58.7%)	χ^2^test=0.18	0.67
Female	14 (45.2%)	166 (41.3%)		
Hypertension				
No	29 (93.5%)	379 (94.3%)	Fisher's exact test=0.03	0.87
Yes	2 (6.5%)	23 (5.7%)		
DM				
No	29 (93.5%)	380 (94.5%)	Fisher's exact test=0.05	0.82
Yes	2 (6.5%)	22 (5.5%)		
Both hypertension and DM				
No	31 (100%)	388 (96.5%)	Fisher's exact test=1.12	0.29
Yes	0 (0.0%)	14 (3.5%)		
Blood types				
A+	18 (58.1%)	132 (32.8%)		
AB	3 (9.7%)	20 (5.0%)		
B+	4 (12.9%)	57 (14.2%)	χ^2^test =12.22	0.06
B-	0 (0.0%)	3 (0.7%)		
A-	0 (0.0%)	9 (2.2%)		
O+	6 (19.4%)	159 (39.6%)		
O-	0 (0.0%)	22 (5.5%)		
Perioperative factors				
ASA classification				
≤2	21 (67.7%)	303 (75.4%)	χ^2^test =0.89	0.35
>2	10 (32.3%)	99 (24.6%)		
Prophylactic antibiotics				
No	18 (58.1%)	279 (69.4%)	χ^2^test =1.72	0.19
Yes	13 (41.9%)	123 (30.6%)		
Preoperative shaving				
No	26 (83.9%)	297 (73.9%)	χ^2^test =1.52	0.22
Yes	5 (16.1%)	105 (26.1%)		
Antiseptic used for skin preparation				
Providone Iodine	10 (32.3%)	166 (41.3%)	χ^2^test =0.97	0.32
Chlorhexidine	21 (67.7%)	236 (58.7%)		
Type of surgery				0.0001*
Open	24 (77.4%)	166 (41.3%)	χ^2^test =15.25	4.11 (1.68-10.06)
Laparoscopic	7 (22.6%)	236 (58.7%)		
Duration of the procedure (min.)	9.71±9.29	3.06±4.13	Mann Whitney U test=6.44	0.0001*
				1.17 (1.08-1.27)
Urgency of the operation				
Urgent	31 (100%)	392 (97.5%)	Fisher's exact test=0.79	0.37
Elective	0 (0.0%)	10 (2.5%)		
Converting to open appendectomy				
No	30 (96.8%)	392 (97.5%)		
Yes	1 (3.2%)	10 (2.5%)	Fisher's exact test=0.06	0.8
Wound infection category				
Clean-contaminated	31 (100%)	400 (99.5%)	Fisher's exact test =0.15	0.69
Contaminated	0 (0.0%)	2 (0.5%)		
Histopathology of the appendix				
No pathology				
Yes	0 (0.0%)	32 (8.0%)	Fisher's exacttest =2.66	0.15
No	31 (100%)	370 (92.0%)		
Simple appendicitis				
Yes	4 (12.9%)	70 (17.4%)	χ^2^test =0.41	0.52
No	27 (87.1%)	332 (82.6%)		
Complicated appendicitis				
Yes	27 (87.1%)	295 (73.4%)	χ^2^test =2.84	0.09
No	4 (12.9%)	107 (26.6%)		
Gangrenous				
Yes	2 (6.5%)	24 (6.0%)	Fisher' exact test =0.01	1
No	29 (93.5%)	378 (94.0%)		
Perforated				0.002*
Yes	9 (29.0%)	36 (9.0%)	Fisher' exacttest =12.46	4.16 (1.78-9.71)
No	22 (71.0%)	366 (91.0%)		
Suppurative				
Yes	16 (51.6%)	231 (57.5%)	χ^2^test =0.40	0.53
No	15 (48.4%)	171 (42.5%)		
Other findings				
Neoplasm	0 (0.0%)	4 (1.0%)	χ^2^test =0.39	0.82
Schistosomiasis	0 (0.0%)	1 (0.2%)		

As for some of the postoperative laboratory investigations that we studied, higher WBC levels with a mean of (15.13 K/µL) and lower albumin levels with a mean of (30.82 g/L) were associated with increased risk for SSI post-appendectomy, p=0.004 and p=0.011, respectively. However, hemoglobin level was not considered to be significantly associated with SSI (p=0.763). Moreover, having postoperative SSI leads to a longer length of stay (p=0.0004), with a mean of nine days, compared to non-SSI cases with a mean of three days only (Table 3).

**Table 3:** Mean values of the statistically significant quantitative factors for SSI post-appendectomy WBCs - white blood cells

Factors	Having SSI	
	Yes	No
Duration of the surgery (min)	117.5	88.0
WBCs (K/µL)	15.13	12.60
Albumin (g/L)	30.82	36.08
Hospitalization time (days)	9	3

## Discussion

In our study, the prevalence of SSI between 2013 and 2017 among post-appendectomy patients reached 7.2% of the total. When comparing this result to different studies, a wild variation in the prevalence of SSI post-appendectomy is found. In two studies in Europe, Petrosillo et al. in 2008 had a total of 5.2% cases of SSI in a multicentric prospective study, while Aranda-Narvaez et al. in 2014 had an overall SSI rate of 13.4% [17,18]. Two other studies in Egypt and Qatar found a rate of 21.9% and 3.6%, respectively [10,19]. A local study revealed the rate of SSI prevalence to reach 3.96% [16].

Many factors have been studied in the literature that can be contributed to the incidence of SSIs. Age, gender, and nationality of the patients were not significant factors for SSI in our study, which is similar to results from other studies on different types of operations [20,21]. Moreover, being male has not shown a significant relationship in this study compared to a study on German national surveillance concluded that gender was considerably related to SSI (p<0.001)[22].

Giving prophylactic antibiotics has been stated as an important risk factor. The number, timing, and type of antibiotic have been well studied. Prophylactic antibiotics are specified to be within 60 minutes before the surgery in our hospital. One hundred thirty-six (31.4%) patients were given prophylactic antibiotics, where 85 (19.6%) received two or more types of antibiotics while 50 (11.5%) used only one type. Cefuroxime was the most used one (45.49%) followed by metronidazole (44.14%) separately. However, a significant relation to SSI was not found in this study except for cefuroxime, which was associated with a lower risk of SSI post-appendectomy. A multicentric Japanese study also assumes that antibiotics do not have a protective role in SSI post gastrointestinal surgeries including appendectomy; therefore, it does not consider as a risk factor for SSI [23].

SSI is strongly affected by the ASA score in patients undergoing clean or clean-contaminated procedures, which has been found by Khan et al., in his study [24], where a significant postoperative infection rate among those with ASA II and ASA III was higher than those with ASA I. And they also concluded a higher rate of infection in patients with co-morbidities including hypertension and diabetes with ASA II and ASA III. All of our patients have been placed within the category of clean-contaminated wounds while only two of them were under the contaminated category. Most of our patients were classified into grades I E, II E, III E, and II, 320 (73.9%), 81 (18.7%), 17 (3.9%), and 5 (1.2%) patients, respectively. In addition, we found 25 (5.8%) hypertensive and 24 (5.5%) diabetic patients. Four (12.9%) of them diagnosed with SSI, but with no significant association.

With regards to the urgency of the operation and its effect on the rate of infection postoperatively, a comparison with Watanabe et al. study has been found a high and significant rate of infection (p<0.001) with patients who were treated in emergent surgery rather than the elective one [20]. However, this significance did not exist in this study because of the low number of patients (2.3% of the total) who did elective appendectomy.

 Intraoperative factors including hair removal at the site of incision and type of skin preparation were not significant for SSI (p=0.22) and (p=0.32), respectively, where preoperative shaving was done only for 25.4% of the patients. And chlorhexidine type of skin preparation was used for 59.4% of the patients. So, we agreed with Watanabe et al., results of preoperative shaving as a factor, by non-significant relation [20]. While a randomized control trial by Darouiche et al., was done on clean-contaminated surgeries disagreed with our result regarding the type of skin preparation, where chlorhexidine was more effective against SSI (p=0.004) [25].

Varela et al. study found a significant relationship between the type of procedure either by a laparoscopic or open appendectomy and getting SSI (p>0.001), where open procedures have a higher risk of getting a postoperative wound infection [26]. Our study supports this result (p=0.0001) where more numbers of all SSIs were found with the open appendectomy approach (77.4%) and this can be explained by the larger size of the incision associated with it. And more reasonable cause, that laparoscopic approach is known to have less hospitalization time. The current study has a mean [SD] of 2.98 [2.89] days, where the hospitalization time among SSI patients was 9.71 [9.29] days comparing to non-SSI patients when they had only 3.06 [4.13] days (p=0.000421).

Among our patients, 11 appendectomies out of 433 were converted from laparoscopic to open surgery, and this factor showed no significant relation with the occurrence of SSI, while a study that was done on children in 2014 showed the opposite (p<0.001) [27].

Duration of the surgery is another important factor, and the literature discussed it widely. A study has been done on post-appendectomy patients showed a significant relation(p<0.0001) [27]. On the other hand, the duration of the appendectomy procedure has no role (p=0.141) with the incidence of SSI in a study by Giesen et al. [28]. Our study emphasizes the first opinion and confirms it (p=0.0001).

Some articles studied the relation between histopathological type of appendix post-appendectomy and the incidence of SSI. In a multicentric study in the Netherlands, 42 out of 637 patients got an SSI after appendectomy [28] in which simple acute appendicitis, perforated appendicitis, gangrenous appendicitis, and purulent appendicitis, constitute 64.7%, 17.3%, 11.3%, and 23.2% of the total number of the cases, respectively. In that study, a significant relation was found between perforated and purulent complex types of appendicitis and SSI (p<0.001). In our study, among the 433 patients, post-appendectomy histopathological assessment showed 32 (7.4%) with no pathology, 74 (17.1%) simple acute appendicitis, 318 (73.4%) complicated appendicitis, four (0.9%) neoplasm appendicitis, one (0.2%) schistosomiasis appendicitis, and four (0.9%) cases with unavailable histopathology report. Similar to the previously mentioned study, a significant relation with SSI was found in the particularly perforated type of appendicitis (p=0.002) [28]. Perforated appendicitis consists of 29.0% of the SSI cases, and unlike that study, suppurative (purulent) type of appendicitis (51.6% of SSI cases) was not significantly associated with SSI, along with other appendicitis with different histopathological findings also.

According to the postoperative WBCs level and their impact as a predictor for SSI, it was studied by Povšič, among colorectal cancer patients and he found no significant relation. Although, low serum albumin is an important risk factor as it affects the general condition of the patient nutritionally and would lead them to a bigger chance to be immunocompromised and therefore for SSI postoperatively [29]. Mujagic et al. also focused on some blood parameters as predictors for SSI where lower levels of hemoglobin and albumin were significantly associated with SSI [30]. In our study, significant results regarding WBCs and albumin level post-appendectomies were found where a higher mean of WBCs (15.13 [5.24] K/µL) and a lower mean of albumin levels (30.82 [9.38] g/L) was associated with positive cases of SSI. And so on, a lower mean of WBCs (12.60 [4.69] K/µL) and a higher mean of albumin levels (36.08 [6.59] g/L) was associated with the negative cases. Anyway, the hemoglobin level was not a significant factor.

This study has certain limitations due to poor documentation, particularly in older years, such as the absence of some laboratory tests (e.g. Hemoglobin and inflammatory marker). Some cultures and sensitivities might not involve a specific organism type. In rare circumstances, the timing of prophylactic administration, particularly in a short time window (60 minutes), maybe imprecise, and prospective studies may be beneficial.

## Conclusions

Controlling the high rate of SSI by using the optimal technique of approach, decreasing the duration of the surgery, and early intervention before reaching a more advanced stage of appendix pathology may help more in reducing SSI post-appendectomy. Taking other preoperative and postoperative factors into consideration will lead to better outcomes for the patients including less hospitalization time and consumption of human and hospital resources.

This paper covered the geographic area and multicenter studies with prospective aspects and larger sample size are recommended and more investigation toward intraoperative and postoperative factors should be assessed in addition to the burden of the infections assurance that affects the health care system.
